# Evidence-based guideline implementation in low and middle income countries: lessons for mental health care

**DOI:** 10.1186/s13033-016-0115-1

**Published:** 2017-01-05

**Authors:** Mary Docherty, Kate Shaw, Lucy Goulding, Hannah Parke, Erica Eassom, Farnoosh Ali, Graham Thornicroft

**Affiliations:** 1 South London and Maudsley NHS Foundation Trust, The Maudsley Hospital, Denmark Hill, London, SE5 8AF UK; 2 Centre for Implementation Science, Institute of Psychiatry, Psychology and Neuroscience, King’s College London, London, UK; 3 Centre for Global Mental Health and Centre for Implementation Science, Institute of Psychiatry, Psychology and Neuroscience, King’s College London, London, UK

**Keywords:** Guideline implementation, Clinical practice guideline, Evidence-based practice, Implementation science, Implementation strategies, Low income countries, Middle income countries, Mental health, Physical health, Systematic literature review

## Abstract

**Background:**

There is a significant treatment gap in provision of effective treatment for people with mental disorders globally. In some Low and Middle Income Countries (LMICs) this gap is 90% or more in terms of untreated cases. Clinical practice guidelines (CPGs) are one tool to improve health care provision. The aim of this review is to examine studies of the effectiveness of evidence-based CPG implementation across physical and mental health care, to inform mental healthcare provision in low and middle income countries (LMICs), and to identify transferable lessons from other non-communicable diseases to mental health.

**Methods:**

A systematic literature review employing narrative synthesis and utilising the tools developed by the Cochrane Effective Practice and Organisation of Care (EPOC) group was conducted. Experimental studies of CPG implementation relating to non-communicable diseases, including mental disorders, in LMICs were retrieved and synthesised.

**Results:**

Few (six) studies were identified. Four cluster randomised controlled trials (RCTs) related to the introduction of CPGs for non-communicable diseases in physical health; one cluster-RCT included CPGs for both a non-communicable disease in physical health and mental health, and one uncontrolled before and after study described the introduction of a CPG for mental health. All of the included studies adopted multi-faceted CPG implementation strategies and used education as part of this strategy. Components of the multi-faceted strategies were sometimes poorly described. Results of the studies included generally show statistically significant improvement on some, but not all, outcomes.

**Conclusion:**

Evidence for the effectiveness of interventions to improve uptake of, and compliance with, evidence-based CPGs in LMICs for mental disorders and for other non-communicable diseases is at present limited. The sparse literature does, however, suggest that multifaceted CPG implementation strategies that involve an educational component may be an effective way of improving guideline adherence and therefore of improving clinical outcomes. Further work is needed to examine cost-effectiveness of CPG implementation strategies in LMICs and to draw conclusions on the transferability of implementation experience in physical health care to mental health practice settings. Strategies to ensure that CPGs are developed with clear guidance for implementation, and with explicit, methods to evaluate them should be a priority for mental health researchers and for international agencies.

**Electronic supplementary material:**

The online version of this article (doi:10.1186/s13033-016-0115-1) contains supplementary material, which is available to authorized users.

## Background

The global burden of mental, neurological and substance use (MNS) disorders is relentlessly high, resulting in long-term disability combined with premature mortality [1, 2]. Untreated MNS disorders also have a negative impact on global health priorities [3], and may be associated with human rights abuses [4]. The majority of people with MNS disorders in LMICs are unable to access effective mental health care, with the treatment gap higher than 90% in many such countries [5–7]. The most recent estimates of the global burden of mental and neurological disorders suggest that these may be considerably greater than previously thought [8].

Issues related to quality improvement and implementation science are central to these challenges [9, 10]. Health system constraints are recognised to be potent threats to the scale-up of access to evidence-based mental health care for people affected by MNS disorders in low- and middle-income countries (LMICs) [11]. Policy makers and planners play a critical role in the successful strengthening of mental health systems, but may not be appropriately equipped for the task. For example, in a mixed-methods study of the challenges faced during implementation of national mental health policy in South Africa, the key barriers included the low priority given to mental health care by planners, provincial bureaucracy around service coordination, insufficient staff for policy-making and service planning, and disinclination by some local authorities to lead mental health policy implementation [12].

In a qualitative study involving national and regional stakeholders in Ghana, South Africa, Uganda and Zambia, low perceived legitimacy of the problem of scaling up mental health services and inadequate government support were identified as factors perpetuating the low priority accorded to mental health care [13]. A survey of leaders and specialists in international mental health specifically identified a need for a more over-arching public health perspective among mental health policy-makers [14]. The lack of training and experience of clinicians to fulfil leadership roles in policy making and planning was particularly emphasised.

There is international consensus on the need for mental health system strengthening and for a specific focus on building the capacity of key stakeholders, including policy makers and planners and service users [15–18].

**Fig. 1:**
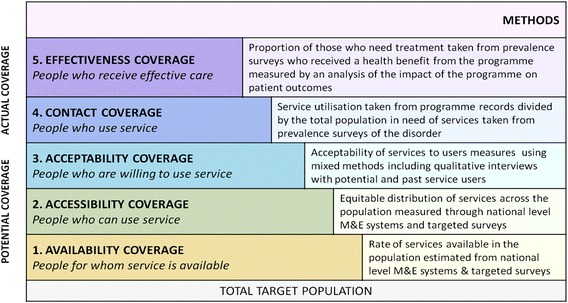
Schema to show different types of treatment coverage [19, 24]

These issues speak to the *quantity* of mental health care available in LMICs. Indeed, the key mental health focus of the WHO is the *mental health gap,* namely the difference between true prevalence rates of mental disorders and treated prevalence rates. Such treated prevalence rates are sometimes expressed as *treatment coverage.* Nevertheless the usual way in which coverage is conceptualised and measured refers to *contact coverage*, i.e. the occurrence of any treatment encounter, whether or not this confers benefit to the patient. A more satisfactory definition refers to quality of care as expressed in terms of *effectiveness coverage*, which means the proportion of people with mental disorders, at a time point or over a time period, who receive effective treatment and care (see Fig. 1) [19].

Regarding implementation, it is clear in terms of the provision of mental health care that there are two central problems: weak or absent (1) national mental health plans, and (2) mechanisms to implement these national plans, including patient and practitioner level interventions such as evidence-based clinical practice guidelines, which are simply not put into practice. One can therefore speak of an *implementation gap,* which is a complex set of barriers standing in the way of better mental health care across most countries of the world [20]. Among the reasons for non-implementation of carefully constructed national level strategic plans, and local level treatment guidelines is the paucity of leadership skills for general health systems strengthening [17, 21–23].

Turning now to the *quality of care*, clinical practice guidelines (CPGs) are an established tool to support reduction in variation, improvement in quality and efficiency, and delivery of evidence-based care [24]. Over the last two decades there has been a relatively large production of mental health guidelines in high-income countries, particularly in the UK, Canada, New Zealand and Australia. With respect to these efforts however, only a small corollary body of research focused on their implementation [25]. Recognition of this knowledge gap and lack of clarity as to the benefits of implementing specific psychiatric guidelines in routine primary care or mental health specialist teams [26] has led to calls for more formal evaluations in this field [27].

A recent systematic review concluded that there was sufficient evidence from high income countries to view CPGs in mental healthcare as an essential asset if appropriately developed and implemented [28]. They showed trends towards improvement in process and patient outcomes following guideline implementation [28]. This supports the legitimacy of CPG development and implementation in LMICs as one of a number of possible tools to approach scale up of mental health care in these settings. On-going difficulties in methodology to evaluate implementation techniques has however been identified as a significant barrier to optimising the potential value of evidence-based guidelines as tools for population level improvements in care [28].

The context for this paper is therefore an appreciation that little has so far been published within the mental health field about implementation of evidence-based policies and practices in LMICs [29–31]. At the same time we wish to learn from other health sectors of how such progress in LMICs can be made. The aims of this paper are therefore to: (1) review evidence for CPG implementation in physical and mental health care settings in LMICs; (2) consider the transferability of lessons learned from implementation of CPGs in physical health to mental health in LMICs.

## Methods

### Design of the review

The systematic review was conducted following guidelines produced by the Cochrane Collaboration [32]. The PRISMA checklist was used to inform reporting of the review [33].

### Searches

The following electronic databases were searched: MEDLINE, EMBASE, PsychInfo, Global Health and LILACS (Latin American and Caribbean Health Sciences Literature). The search strategy was prepared for MEDLINE (see Additional file 1: Web Appendix) and then translated to other databases following their requirements. Literature published since the earliest date indexed in each database up to the search date (October 2014) was retrieved. Duplicates were removed prior to screening.

Reference list checks of studies found during the electronic search were made. General searches were conducted using internet search engines and key authors in the field were asked to indicate potentially relevant studies. The review team did not have any funds to provide translation; however, Google Translate was used to translate the full text of three studies that were not reported in English. None of these papers met the inclusion criteria.

### Study inclusion and exclusion criteria

#### Design

Studies relating to a non-communicable physical health condition using any of the following designs were included: randomised controlled trials (RCT), cluster-RCTs, controlled clinical trials (CCT), controlled-before-and-after studies (CBA) and interrupted time series (ITS) studies with concurrent controls.

Studies relating to a mental health condition using any of the following designs were included: randomised controlled trials (RCT), cluster-RCTs, controlled clinical trials (CCT), controlled-before-and-after studies (CBA), interrupted time series (ITS) studies with concurrent controls, and uncontrolled before and after studies.

Included studies were permitted to have any number of sites. Studies with non-experimental designs were excluded. However, findings from process evaluation or qualitative studies conducted alongside experimental studies were included in the data extraction and synthesis of results.

#### Countries and populations included

Included studies were conducted in a low or middle income country. The World Bank Atlas method for 2014 defined low-income economies as those with a GNI per capita of $1045 or less; middle-income economies had a GNI per capita of more than $1045 but less than $12,746. Studies conducted in high-income countries were excluded.

Eligible participants in included studies were children or adults of any age with a non-communicable disease—including mental and physical health conditions. Examples include: depression, dementia, coronary heart disease, cancer. Studies solely involving participants with communicable diseases (e.g. malaria, HIV, influenza) were excluded. Studies involving outcomes related to surgical site infection and hand hygiene were also excluded. Studies that included participants with both non-communicable and communicable diseases were included with outcomes for people with non-communicable diseases being the focus during synthesis and reporting of the results.

#### Interventions

Included studies described the introduction of a CPG relating to management of people who have a non-communicable disease (including mental and physical health). The Institute of Medicine’s definition of a CPG was adopted. This states that “clinical practice guidelines are statements that include recommendations intended to optimize patient care that are informed by a systematic review of evidence and an assessment of the benefit and harms of alternative care options” [34].

Studies that assessed the implementation of more than one CPG were included if at least one of the guidelines was in relation to a non-communicable disease (physical or mental health); and results were drawn from that part of the study only. CPGs produced for a health system, a group of healthcare professionals, a country, state, or province were included. Included studies described and assessed the guideline implementation strategy adopted. No restrictions were placed upon the type or number of strategies used. Studies that did not describe a guideline implementation strategy were excluded.

#### Comparisons

For studies targeting physical health conditions, included studies could compare a group where one or more CPG implementation strategies was used to a control group where no specific implementation strategy was adopted, or groups using different implementation strategies could be directly compared.

For studies targeting mental health conditions, included studies did not have to include a control group. In such cases, the comparison of interest was outcomes before and after the introduction of the CPG and supporting implementation strategy. For study designs employing control groups, included studies could compare a group where one or more CPG implementation strategies was used to a control group where no specific implementation strategy was adopted, or groups using different implementation strategies could be directly compared.

#### Outcomes

Included studies demonstrated pre and post measurement of processes or outcomes targeted by the CPG. Outcome measures could therefore concern the effectiveness of the implementation strategy measured by compliance with the CPG (for example prescribing behaviours), changes in the knowledge, attitudes, beliefs of behaviours of healthcare professionals, or changes in patients’ health. Outcomes could be clinician or patient reported. Post-implementation measurement could take place at any point following the introduction of the CPG and accompanying implementation strategies. Studies that did not include pre and post CPG implementation measurement were excluded.

### Screening

Screening of titles and abstracts and full-texts was conducted by four authors (MD, KS, HP and EE). Authors met to agree on final inclusion. Uncertainties experienced during the screening and extraction process were resolved with input from an additional author (LG).

### Data extraction and quality assessment

Three authors (MD, KS and FA) independently extracted data from included studies and appraised the potential risk of bias in each study using tools developed by the Cochrane Effective Practice and Organisation of Care (EPOC) group [35]. An additional author (LG) double checked all data extractions and risk of bias assessments. The Cochrane Collaboration’s ‘EPOC taxonomy’ was used to classify the implementation strategies adopted in included papers [36].

### Data synthesis

Due to the nature of the review question there was heterogeneity in the participants, interventions, comparisons, outcome measures and outcomes of included studies. A descriptive data synthesis was consequently undertaken to summarise the characteristics and results from included studies in table form and to address the review questions.

## Results

### Results of the search

**Fig. 2:**
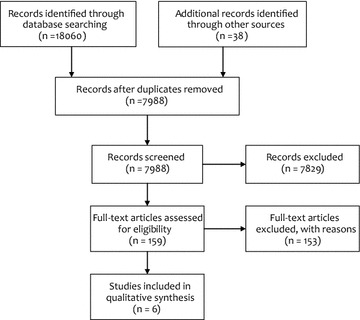
PRISMA flow diagram of inclusion and exclusion process [33]

The electronic searches yielded 18,060 citations. 7950 remained after duplicates were removed. An additional 38 citations were uncovered by hand searching reference lists of key papers and through contact with experts in the field. This was reduced to 159 papers for full text screening, of which 6 met the inclusion criteria for the review. Details of included studies are provided in Table 1 and Fig. 2.

**Table 1:** Description of studies meeting the inclusion criteria

Lead author (year of publication) Country Mental or physical health	Study design Risk of bias assessment	Health condition or behaviour targeted by Clinical Practice Guideline (GPG) Setting and participants	Brief description of intervention / control conditions and implementation strategies adopted (classified according to EPOC taxonomy)	Primary outcomes	Narrative summary of results
Berwanger et al. [37] Brazil Physical health (acute coronary syndromes)	Cluster randomised trial Low–moderate risk of bias	*CPG* The intervention included posters containing evidence-based recommendations for the treatment of acute coronary syndromes (ACS) *Setting and participants* 36 general public hospitals with emergency centres in major urban areas in Brazil. 19 hospitals were randomised to receive the intervention and 17 to control. The analysis represented 602 patients with a diagnosis of ACS in the intervention group and 548 in the control group. Detail regarding the number and type of health professionals exposed to the intervention is not provided	*Intervention* A multifaceted educational quality improvement intervention for management of acute coronary syndromes Implementation strategies adopted were: reminders; case management; educational materials; educational outreach; local opinion leaders Checklists were also used Control: routine care	The percentage of eligible patients who received all appropriate evidence-based therapies (aspirin, clopidogrel, anticoagulants and statins) within the first 24 h of admission to hospital without contraindications	A multifaceted educational intervention resulted in statistically significant improvement in the use of evidence-based therapies both within 24 h of admission and at discharge in comparison to routine care There were no statistically significant differences in the rate of major cardiovascular events, mortality at 30 days, new myocardial infarction or incidence of major bleeding—however, the study was not powered for evaluation of clinical outcomes Adherence to the reminders and checklists by healthcare professionals in the intervention group was 82.7%
Du et al. [52] China Physical health (acute coronary syndromes)	Cluster randomised controlled trial Moderate-high risk of bias	*CPG* Clinical pathways for acute coronary syndromes based on the American Heart Association / American College of Cardiology guidelines *Setting and participants* 70 urban hospitals throughout China routinely admitting >100 patients annually with suspected acute coronary syndromes were randomly allocated to receive the intervention (n = 32 hospitals, n = 8049 patients) or control (n = 38 hospitals, n = 5731 patients) Detail regarding the number and type of health professionals exposed to the intervention is not provided	*Intervention group* Hospitals implemented clinical pathways (and accompanying multifaceted implementation strategies) for acute coronary syndromes ‘early’ Implementation strategies adopted: audit and feedback; monitoring the performance of the delivery of healthcare; educational meetings *Control group* Usual care for first year. Hospitals implemented clinical pathways (and accompanying implementation strategies) for acute coronary syndromes ‘late’ (1 year after intervention sites) Key performance indicators in intervention sites were collected 12 months after the introduction of the intervention and compared with baseline data in control sites The guideline was tailored to the local context. Teams led by senior cardiologists took responsibility for implementation	Proportion of patients with final diagnosis consistent with biomarker findings; pro- portion of patients with ST-segment-elevation myocardial infarction (STEMI) receiving thrombolysis or primary percutaneous coronary intervention (PCI) among those arriving within 12 h of symptom onset; door-to-needle time for patients with STEMI undergoing thrombolysis; door-to-balloon time for patients with STEMI undergoing primary PCI; proportion of high-risk patients undergoing coronary angiography; proportion of low-risk patients (no on-going symptoms, persistently normal electrocardiogram, and persistently normal biomarkers) undergoing functional testing; proportion of patients discharged on combination medical therapy (including any antiplatelet therapy, β-blocker, angiotensin-converting enzyme inhibitor or angiotensin receptor blocker, and statin); length of stay	The use of a clinical pathway for treatment of acute coronary syndromes compared with usual care statistically significantly improved use of secondary prevention treatments (an increase in the number of patients who were discharged on appropriate medical therapy) but there were no statistically significant differences on other measures of quality of care A survey of 556 health professionals found that 98.2% had heard of the intervention, and >80% had attended training sessions, used the pathway in clinical practice and were aware of study reports.>90% completely or strongly agreed that the pathway was valuable. A qualitative process evaluation conducted alongside the trial aids understanding of findings
Okasha et al. [42] Egypt Mental health	Uncontrolled before and after study High risk of bias	*CPG* The tenth revision of the International Statistical Classification of Mental and Behavioural disorders Primary Health Care (ICD-10 PHC) Setting and participants: 20 GPs working in 6 primary health care centres (rural and urban) in Egypt	Intervention: Training was conducted by an experienced psychiatrist. The ICD-10 training kit, an educational training programme and materials produced by the WHO Implementation strategies therefore comprised: educational meetings and educational materials	GP’s attitudes, knowledge, interview skills and ability for diagnosing psychiatric disorders in a primary care setting	There was statistically significant improvement in GP’s optimism about helping patients with mental disorders and in their confidence in their ability to diagnose mental disorders following the intervention. There was no statistically significant change in GP’s interest in patients with mental disorders or in their reporting of the importance of diagnosing mental disorders.
Pagaiya and Garner [41] Thailand Physical health (diabetes mellitus) and mental health (anxiety and panic disorder)	Cluster randomised controlled trial Moderate risk of bias	*CPG* Four clinical guidelines introduced: two for children (acute respiratory infection and diarrhoea); two for adults (diazepam prescribing for anxiety and panic disorder and management of diabetes mellitus) *Setting and participants* Health centres in Khon Kaen province in Thailand, mainly in rural areas. 9 health centres were randomised to receive the intervention and 9 to control. 110 patients for diazepam prescribing and all diabetes patients at each centre were included	*Intervention group* introduction of four clinical guidelines (two of which meet the inclusion criteria for the present review) plus the following implementation strategies: educational meetings; educational out-reach visits; audit and feedback *Control group* usual care. The PRECEDE-PROCEED implementation framework was adopted	Diazepam prescribing; prescribing costs per patient; management of diabetes	Clinical guidelines implemented with educational meetings and outreach visits and audit and feedback improved some aspects of prescribing but not others in the short-term Diazepam prescribing was statistically significantly reduced in the intervention group. There was no statistically significant difference in the management of diabetes between the intervention and control groups. For all four CPGs combined, average drug costs per patient in the intervention group were statistically significantly less than in the control group
Shrestha et al. [39] Nepal Physical health (asthma, COPD)	Cluster randomised trial High risk of bias	*CPG* Practical Approach to Lung health (PAL) guidelines on prescription behaviour and total cost of prescription for patients with asthma, pneumonia and chronic obstructive pulmonary disease (COPD) Setting and participants: Of 76 health facilities in Nawalparasi district, 40 were included in the study on the basis of highest patient attendance. Facilities were stratified by type and subsequently randomized to receive the intervention [21] or usual practice control [19]. Detail regarding the number of patients in each group is not provided, rather number of prescriptions is given. Detail regarding the number and type of health professionals exposed to the intervention is not provided	*Intervention* Introduction of the PAL guideline plus the following accompanying implementation strategies: educational materials; educational meetings; educational outreach (train-the trainer) *Control* Usual practice The guideline was adapted to the Nepalese context. Different professionals, organisations and health workers were involved in this process	Healthcare professional prescribing practices for asthma and COPD	The guidelines and accompanying implementation strategies led to a statistically significant reduction in poly-pharmacy (multiple prescriptions) in comparison to the control condition. There were no statistically significant differences in the prescription of generic and essential drugs, average prescription cost and wastage cost, or prescription of antibiotics between the intervention and control groups
Steyn et al. [40] South Africa Physical health (diabetes mellitus and hypertension)	Cluster randomised controlled trial Low -moderate risk of bias	*CPG* A structured record which incorporated the national guidelines for the management of patients with type 2 Diabetes mellitus or hypertension or both conditions *Setting and participants* 18 randomly selected public sector primary healthcare clinics known as Community Health Centres (CHCs) in Cape Town in 1999 and 2000. 9 clinics were randomised to deliver the intervention and 9 to control. At baseline, in the intervention clusters there were n = 229 patients with diabetes and n = 461 patients with hypertension. At baseline, in the control clusters there were n = 227 patients with diabetes and n = 459 patients with hypertension. The intervention was targeted at General Practitioners and nurses who consult patients with chronic disorders. Detail regarding the number of health professionals exposed to the intervention is not provided	*Intervention* a structured record that included algorithms for diagnosis and guideline-based management of diabetes and hypertension, including educational topics to be covered with the patient Implementation strategies adopted: case management; reminders; educational outreach from a local diabetes and hypertension expert *Control group* usual care, which included the guidelines passively disseminated by the National Department of Health	Mean level of glycated haemoglobin in all patients with diabetes Mean systolic and mean diastolic BP in all those with hypertension as measured at the end of the 1 year intervention period	The intervention had no statistically significant impact on either diabetes or hypertension control No differences were observed in the recording of process measures, e.g. visual acuity or foot examinations The process measures collected implied that the structured record was not widely adopted by healthcare professionals in the primary care clinics. At follow-up, the structured record was found in the folders of only 58% of patients with diabetes and 47% of patients with hypertension

#### Included studies

Six papers met the inclusion criteria for the review. Four of these papers related to the introduction of CPGs for non-communicable diseases in physical health [37–40], one paper included CPGs for both a non-communicable disease in physical health and mental health [41] and one paper examined the introduction of a CPG for mental health [42].

#### Design

Five cluster randomised controlled trials (cluster RCTs) [37–41] and one uncontrolled before and after study (in mental health) [42] were included in the review.

#### Population

Due to the nature of the interventions being reviewed (implementation of CPGs); all interventions were delivered to healthcare professionals. Where specified, participants receiving the intervention were typically physicians and nurses.

#### Setting

Included studies were conducted in Brazil [37], China [38], Thailand [41], Nepal [39], South Africa [40] and Egypt [42]. Two studies were set in general hospitals in urban areas [37, 38]. Four studies were set in primary health care centres: one of these was conducted in an urban area [40]; two in rural areas [39, 41] and one in a mix of urban and rural areas [42].

#### Behaviour/clinical condition targeted by the CPG

The non-communicable clinical conditions and behaviours targeted by the CPGs were as follows: use of evidence-based therapies for acute coronary syndromes [37, 38]; diazepam prescribing practices for anxiety or panic disorder and management of diabetes mellitus [41]; prescribing practices for asthma and COPD [39]; management of diabetes mellitus and hypertension [40]; and management of mental and behavioural disorders in primary healthcare through implementation of the ICD-10 [42].

#### Format of clinical practice guidelines

Evidence-based guidelines were presented by: poster [37]; a clinical pathway document containing structured algorithms to be filled in by the healthcare professional [38]; a structured record to be filled in by the healthcare professional which was a three sided, folded A3 sheet of coloured paper placed with each patient’s notes [40]; and A4 laminated documents printed on coloured paper [41]. In two studies, the format of the CPG was unclear [39, 42]. Four studies report that the CPG was either originally developed for the local context or tailored to the local context [38–41].

#### CPG implementation strategies adopted

All of the included studies adopted multi-faceted CPG implementation strategies. The components of the multi-faceted interventions were sometimes poorly described. All studies employed implementation strategies that targeted healthcare professionals’ use of the CPG. Implementation strategies adopted to facilitate uptake of CPGs (classified according to the EPOC taxonomy) were: reminders, case management, educational materials and educational outreach [37]; audit and feedback, monitoring the performance of the delivery of healthcare and educational meetings [38]; educational meetings, educational out-reach and audit and feedback [41]; educational materials, educational meetings and educational out-reach [39]; case management, reminders and educational outreach [40]; educational meetings and educational materials [42].

Only one study [41] explicitly described adoption of a theoretical framework to guide CPG implementation. One paper reported the lack of adaptation of the CPG implementation strategies to the local context as a limitation [40]—this finding was pronounced in qualitative interviews undertaken with healthcare professionals as part of a process evaluation.

### Control groups

In four of the cluster-RCTs, the intervention (CPG and accompanying implementation strategies) was compared to routine care (no CPG or accompanying implementation strategies) [37–39, 41]. In one cluster-RCT, the intervention (CPG and accompanying implementation strategies) was compared to passive diffusion of the CPG (no accompanying implementation strategies) [40]. The before and after study in mental health [42] did not include a control group.

### Primary outcomes

Primary outcomes relating to processes undertaken by healthcare professionals were: the proportion of patients who received evidence-based treatments for acute coronary syndromes [37, 38] and prescribing practices [39, 41]. Primary outcomes relating to healthcare professionals were: attitudes, knowledge and skills [42]. Primary outcomes relating to patients were level of glycated haemoglobin in patients with diabetes and systolic and diastolic blood pressure in patients with hypertension [40].

### Risk of bias in included studies

The risk of bias in included studies was mixed. In the case of the cluster-RCTs, the nature of the intervention (CPG) and accompanying implementation strategies (e.g. educational sessions) often meant that it was not feasible to blind participants or personnel to the allocation of each cluster. In some cases, staff from different groups met occasionally leading to opportunity for contamination. Randomisation processes were judged to be low risk in four studies [37, 38, 40, 41] and unclear in one study [39]. Risk of bias in the mental health paper that used the ICD-10 [42] was judged to be very high due to the use of an uncontrolled before and after study design.

### Effects of interventions

Results of the included studies generally show statistically significant improvement on some but not all outcomes with the exception of one study [40] where the CPG intervention did not demonstrate any statistically significant improvement in comparison to control. The authors of this paper proposed that poor implementation of their intervention and accompanying implementation strategies was the major contributor to ineffectiveness.

Multifaceted implementation strategies were generally deemed to be effective in encouraging use of guidelines and creating positive change in outcomes. However, it is not possible to infer which approach to guideline implementation is most effective. Furthermore, the small number of included studies means that there are no observable trends according to clinical setting or topic addressed by the CPG (e.g. physical versus mental health).

### Implementation outcomes

Implementation outcomes, as defined by Proctor et al. [43], were infrequently studied. Cost effectiveness of the interventions (e.g. taking into account the cost of delivering the CPG implementation strategies) was not assessed in any of the included studies. However, the effect of the intervention on prescription costs was calculated in two studies [39, 41]. Fidelity and acceptability of the intervention was explored via qualitative process evaluation in two studies [38, 40]. The length of time to follow-up in included studies was generally short: 30 days [37]; 6 months [41]; 8 months [39]; 1 year [38, 40] and unclear but assumed to be immediately following the intervention [42]. This limits the ability to draw inferences regarding sustainability of any improved practice.

## Discussion

The aims of this review were to examine studies of the effectiveness of evidence-based clinical practice guideline implementation for non-communicable diseases in LMICs, and to learn transferable lessons from other non-communicable diseases to mental health. The review revealed a significant paucity of good quality controlled studies not only in the field of mental health but across other non-communicable diseases in LMICs.

The small number of included papers made it difficult to draw clear conclusions about the role of different implementation strategies to support CPGs in mental health or in other non-communicable disease areas. Our inclusion criteria for mental health studies permitted uncontrolled designs in order to capture any existing work in this area. For other non-communicable disease areas, only high quality studies including control groups were included to support better validity of any conclusions being drawn about transferability of lessons learned across health sectors. As a result of this design, the relatively stringent inclusion criteria led to a large number of non-controlled studies in physical health being excluded from review. Future reviews may therefore wish to relax the inclusions criteria to gather a wider range of information which can create a conceptual map of this type of evidence.

This larger volume of excluded non-controlled studies in physical health had significant methodological limitations in addition to the absence of a control group. A number of studies described interventions with adequate study design for inclusion, but did not detail or refer to an evidence-base in the development of the CPG (e.g. [44]). Other common omissions included failure to clearly describe the implementation strategy used, to consider reproducibility of results, or to consider a theoretical framework in which to seat the implementation study and evaluation. Many excluded papers relied on pre and post analysis without clear attention to confounders that may have affected whether the implementation strategy itself, or other variables, impacted upon any observed change in practice. This difficulty is noted ten years after this problem had already been highlighted in a previous literature review [45]. Overall, the methodological limitations of the excluded studies (see Additional file 1: Web Appendix) mirrored similar problems reported within current literature on guideline implementation in high-income countries suggesting that these study design difficulties are consistent across these different health care settings [24, 27, 47].

Most of the included studies did not explore the relative efficacy of one implementation strategy compared to another in supporting the uptake of and adherence to CPGs, leaving important knowledge gaps with regards to the most effective and efficient strategies to support change within low resource settings. Additionally, there was a frequent lack of delineation between the effect of a strategy to improve uptake of guidelines distinct from whether improved implementation actually improved clinical outcomes targeted by the CPG (e.g. [40, 46]).

Multifaceted interventions with an educational component appear to be effective at supporting change, and attention to their delivery alongside CPG implementation is important to ensure optimum impact. Development of the CPG with consideration of local context, including staff attitudes and available resources, appear to be very important. The one study that showed no improvement in any targeted outcomes had developed CPGs specifically for the local context but had not sufficiently developed a feasible implementation strategy. Qualitative interviews with the professionals involved in implementing CPGs in this study revealed not only an ambivalence towards the perceived utility of the intervention, but also a fundamental failure of the guideline to accommodate resource limitations meaning that were the guideline to be fully implemented it would simply not be affordable [40]. In comparison, the studies that tailored both CPG development and implementation to the local context appeared to be relatively more efficacious in respect of achieving targeted outcomes [38–40].

Known barriers to improving mental health provision in LMICS includes the perceived lack of importance of this field relative to other clinical areas. These observations together support the impression that guideline implementers need to engage those using or impacted upon by the CPGS prior to implementing other strategies focused directly on the guideline use. In mental health this could for example include preliminary anti-stigma interventions. Pagaiya recommends that a “well-planned stepwise process be adopted which takes account of both theoretical and empirical evidence, as well as obstacles to change in relation to the individual staff and the local context” in order to approach successful implementation [41].

Prescribing featured in several of the included studies including one of those addressing mental health guidelines (the ICD-10), which may reflect the relatively more straightforward task of monitoring this sort of intervention. It could be argued that other mental health interventions such as the delivery of psychological therapy or case management approach are more challenging to formulate into guidelines and associated guideline adherence monitoring. Medication-based interventions in mental health CPGs implemented in high income countries may be a feasible place for future researchers to gain insight. Consideration of local availability of specific medications, facilities to store them and resource constraints on formulations offered will be essential in developing and implementing such guidelines in lower income settings.

There was an insufficient number of studies to be able to draw conclusions about transferability of findings to specific diseases, populations or care settings. However, the diversity of the included studies illustrates that CPGs can be implemented and evaluated across a range of populations and care settings within LMICs. Asthma, COPD, hypertension, acute coronary syndrome (ACS) and diabetes were targeted in the included studies on physical health conditions and primary care mental health provision and anxiety disorders targeted in the mental health paper. The transferability of lessons from most of the included physical illnesses to mental health seems feasible in principle due to the parallels between these disease courses and treatment requirements. Most conditions studied were chronic diseases which present with a risk of fluctuations and requirement of a series of interventions to support recovery and stability, in keeping with the clinical course of many mental health disorders (e.g. schizophrenia). ACS is an acute presentation with the requirement of treatment intervention and systematic follow up for a period in keeping with most mental health crises (e.g. suicide plans). There were insufficient studies to explore the question of whether similar pathways and protocols can be constructed for mental health conditions but the findings did not exclude the transferability of these approaches.

Despite these similarities in principle supporting the transferability of implementation experience across mental health and other non-communicable disease areas, there are also important differences in these fields. Mental health workers employ different diagnostic processes, with less reliance on technology and more reliance on human resources to deliver both assessment and treatment. These features highlight a risk for even greater variation in practices and the need to learn from experience in other non-communicable disease areas of the importance of embedding systems for standardisation and measurement of interventions within guidelines. Outcomes in mental health are frequently qualitatively different to those in other non-communicable diseases and less amenable to conventional measurements. In the absence of biomarker outcome measures the importance of developing and incorporating simple and tractable measures such as quality of life, ability to sustain employment, activities of daily living (ADLs) or relationships in order to validate and monitor the efficacy of CPGs in this field is essential.

Local context including prevalent knowledge, behaviours and attitudes towards mental health conditions has predicted larger impact on the potential success of a guideline than in many of the non-communicable diseases considered in this review. Despite some literature acknowledging the context in which a guideline was to be implemented, few gave consideration to the range of barriers that would need to be considered in mental health before implementing and evaluating change. Although beyond the scope of this paper, an important area to consider in future reviews is how impediments to changes in practice due to stigma in some communicable disease areas, for example HIV, have been overcome and addressed in guideline development and implementation.

A consistent difficulty observed in included studies was the limited follow up to evaluate longer-term impact and interventions needed to embed changes in clinical practice in the medium and long term. Longer follow up periods are needed to understand the requirements for sustained change such as on-going interventions (e.g. educational updates) or wider system changes (e.g. changes in job roles, informatics or care pathways and checklists). There is evidence that active on-going efforts to support changes in practice may be required beyond the initial implementation period (e.g. [45, 48, 49]. This has important resource implications for health planners looking to embed improvements into routine clinical care. None of the included studies conducted cost effectiveness analyses of CPG implementation which is a very important omission given the resource constraints in these settings. This should be considered a clear priority in future study designs.

Despite most of the included studies showing improvements in a selection of outcomes, it is not possible to reach conclusions regarding the sustainability, feasibility or practicality of these approaches for mental health planners. For example, all included studies used educational implementation strategies and showed them to be broadly efficacious but they can be labour intensive. One difficulty in providing such strategies in LMICs is the available modes and associated resources for delivering the educational intervention. Furthermore, education is an important tool to support changes in clinical practice but rarely a one off solution to altering knowledge and behaviours. Multiple sessions and top ups are usually required alongside rolling programmes to support staff turnover. Implementing interventions based around education usually require additional strategies such as rigorous systems for monitoring to support change. Outreach approaches were used in some of the studies but again these can be costly and if practitioners are geographically very far apart, this would make educational meetings difficult. Educational sessions also reduce time to engage in clinical activity in already resource stretched services, this places an onus on organisers to ensure high quality sessions or material to support perceived utility and buy in towards the CPG.

Another implementation strategy commonly used in low resource settings, known as task shifting, was not adopted in the studies included within this review. Task shifting refers to the process of transferring a task usually delivered by a scare resource such as a physician to a more rapidly trained and less scare resource such as a health care worker. Evidence has shown that this strategy has been effective in increasing use of treatment guides and protocols in the management of a range of non-communicable conditions such as asthma, hypertension, epilepsy, diabetes and depression [50, 51]. Integrated protocols that involved strategies such as care bundles or care pathways that supported whole systems change seem potentially more promising than focus on just the guideline alone (e.g. [52]). Directed studies to explore the use of task shifting alongside other CPG implementation strategies may be an important area for future research in LMICs considering the need for CPGs not only to improve patient outcomes but also to address efficiency.

Despite the emergence of some lessons for mental health planners, the volume of literature excluded from this review (see Additional file 1: Web Appendix) reveals significant limitations in both study methodology and reporting practices and a need to increase the volume of good quality research. This highlights the importance of a concerted effort within LMICs to improve the rigor of CPG implementation studies. Efforts to increase the availability and existence of context adapted evidence-based mental health guidelines are in process but it is essential that mental health care planners and researchers learn from the limitations encountered in other non-communicable disease guideline implementation research. The World Health Organization mhGAP Intervention Guide, for example, is now in use in over 90 countries worldwide, where the guidelines are intended to be locally adapted for each country and each context, but as yet few evaluations of its use have been published [29, 30, 53–55].

This raises the more fundamental question of whether the scope of this review was too narrow. Clearly this field is not at the stage where many RCTs have been published, from which strong summary findings can be draw. Indeed, given the relative infancy of this field, we did consider whether to conduct a broader narrative review of the literature, for example to summarise what is known of barrier and facilitator factors in guideline implementation in LMICs. Similarly we considered including process evaluation papers in this review. On balance we decided to take a narrower focus on the better quality papers for this paper. The results indicate that future reviews may need to adopt somewhat broader criteria, for example including non-experimental studies, until such time as the quality of the available evidence improves sufficiently.

The overall disconnection which was observed between the volume of literature on guideline development in LMICs, and that on guideline implementation raises important questions for those developing and adapting guidelines in mental health about with whom the responsibility lies for ensuring validation and implementation studies of CPGs are conducted. It has been argued that sound validation studies should be considered a prerequisite for conferring the label of an evidence-based mental health guideline [26]. The importance of this approach in the field of mental health in lower resource settings has particular resonance due to the highly variable existing health care resources and infrastructures in which mental health care can be delivered and the requirement of a local approach to map knowledge, attitudes and behaviour before developing an implementation strategy. In resource limited settings with such significant diagnosis and treatment gaps, efforts to increase effective coverage of mental health care must consider implementation strategies and attention to resource constraints as necessary components of the guideline development process.

This review demonstrates the salience of the implementation gap across medical specialities in LMICs and the real risks for mental health in not responding to these lessons from CPG development over the last few decades. Those looking to scale up mental health in LMICs must prioritise implementation research. Those facilitating the development of CPGs, including professional bodies contributing to them, have a responsibility to ensure that their efforts and the money invested in them lead to tangible improvements in care. Unless agreement is made as to how to take forward this requirement for mental health provision, a real opportunity to learn from lessons in other sectors and settings will be missed.

## Conclusions

Current evidence for the effectiveness of interventions to improve uptake of and compliance with evidence based guidelines in LMICs for mental disorders and for other non-communicable diseases is very limited. The literature suggests that multifaceted CPG implementation strategies that involve an educational component may be an effective way of improving guideline adherence and therefore improving clinical outcomes. Further work is needed to examine cost effectiveness of CPG implementation strategies in LMICs and to draw conclusions on the transferability of implementation experience in other non-communicable disease areas to mental health. Strategies to ensure that CPGs are developed with clear guidance for implementation and methods to evaluate them should be a priority for mental health researchers and for international agencies.

## Additional file

**Additional file 1: Web Appendix.** Excluded papers.

## Abbreviations

ACS: acute coronary syndrome
COPD: chronic obstructive pulmonary disorder
CPGs: clinical practice guidelines
EPOC: Cochrane Effective Practice and Organisation of Care
CBA: controlled-before-and-after studies
CCT: controlled clinical trials
GNI: gross national income
ITS: interrupted time series
LMICs: Low and Middle Income Countries
PRISMA: preferred reporting items for systematic reviews and meta-analyses
RCTs: randomised controlled trials
