# Supplementary material for: Evidence-based guideline implementation in low and middle income countries: lessons for mental health care
Source: Int J Ment Health Syst. 2017 Jan 5;11:8. doi: 10.1186/s13033-016-0115-1 (PMC5217244; doi:10.1186/s13033-016-0115-1)
Supplement: Supplementary file 1 — Additional file 1: Web Appendix. Excluded papers. [file 13033_2016_115_MOESM1_ESM.doc]

**Web Appendix 1. Excluded papers**

| Author | Year | Reason for Exclusion |
| --- | --- | --- |
| Bailey et al  Bailie et al  Bailie et al  Bain et al  Baker et al  Balsara et al  Bampton et al  Banjeree et al  Barcelo et al  Basit et al  Belaid and Ridde  Beran et al  Beran et al  Berglund  Bernai et al  Bernardo  Berra et al  Berti et al  Bordon et al  Boskabady et al  Boucar et al  Bouchet et al  Bouchlaka et al  Boulet et al  Brooks et al  Brosseau et al  Brusamento et al  Casey et al  Cavalli-Sforza  Cheah  Cho et al  Chowcheun et al  Cook et al  Dantas et al  de Carvalho  de Souza  Deen et al  Deen et al  Deng et al  Drost et al  du Mortier  Echeverri et al  English et al  Falbo et al  Fang et al  Franco et al  Fuzinatto et al  Gill et al  Goldhaber-Flebert  Govender et al  Gulliford et al  Gulmezoglu et al  Ider et al  Irimu et al  Irimu et al  Jamsek  Jin et al  Jordans et al  Joyner et al  Khrishnaswamy  Kan et al  Karrowni et al  Kathawaroo et al  Kengne et al  Klein et al  Kongnyuy et al  Kuhne et al  Kupczyk et al  Kwast et al  Lalonde et al  Latham et al  Lima et al  Liwsrisakun et al  Loch et al  Lopes et al  Lundgren et al  Luz et al  Moran et al  Mottaghipour et al.  Mousavi et al  Mshelia et al  Mwakyusa et al.  Mwaniki M.K. et al.  Mwaniki P. et al.  Mwita et al.  Naimoli et al.  Nausheen et al.  Navarro et al.  Navipour et al.  Nzinga et al.  Nsimba  Nutley et al.  Obua et al.  Oliver-Vazquez et al.  Onat et al.  Ostojic et al.  Omaswa et al.  Osur et al.  Ozgun et al.  Patel et al.  Paulik et al.  Pedalini et al.  Peng et al.  Penny et al.  Perez Villasante et al.  Perichart-Perera et al.  Phassarang et al.  Phed-on et al.  Phuc et al.  Pinto et al.  Prata et al.  Pumprueg et al.  Queiroz et al.  Rahimi-Rad et al.  Rahman et al.  Ramezankhani et al.  Ramírez Prada et al.  Ratanalert et al.  Rawat et al.  Riachy et al.  Ribeiro et al.  Ristić et al.  Robert et al.  Ross-Degnan et al.  Rowe et al.  Rowe et al.  Saval et al.  Schilperoord et al.  Sharif-Kashani et al.  Sheik et al.  Silva et al.  Siow et al.  Smith et al.  Stanback et al.  Sun & Stewart  Tan & Aït-Khaled  Ten Asbroek et al.  Thamlikitkul et al.  Thiam et al.  Tohtubtiang et al.  Trap et al.  Trostler et al.  Van de Vijver et al.  Verdeli et al.  Verdeli et al.  Visser et al.  Wahlstrom et al.  Weinburg et al.  Xiao et al.  Xu et al.  Xu et al.  Yang et al.  Yu et al | 2010  2007  2004  1997  2012  2009  2007  2011  2010  2014  2012  2010  2006  2010  2008  2008  2007  2008  2004  2008  2014  2002  2009  2012  2012  2012  2012  2009  2005  2000  2010  2003  2007  2002  2012  2014  2003  2003  2014  2010  2005  2009  2011  2009  2012  2011  2013  2012  2009  2012  1999  2004  2012  2012  2014  2013  2011  2010  2012  2008  2012  2010  2004  2009  2001  2009  2013  2010  1996  2012  2012  2009  2005  2013  2014  2012  2014  2012  2010  2013  2013  2006  2014  2014  2013  2006  2013  2012  2011  2009  2007  2014  2004  2002  2003  2012  1997  2013  2010  2010  2002  1993  2014  2005  2008  2009  2002  2011  2009  2013  2012  2007  2005  2010  2008  2011  2011  2007  2013  2010  2011  2010  2007  1996  2009  2012  2013  2008  2010  2011  2013  2009  2004  2007  2007  2006  2005  2004  2012  2005  2001  2013  2012  2008  2003  2004  2003  2001  2013  2014  2013  2014  2015 | Design  Population  Population  Population  Design  Communicable disease  Population  Communicable disease  Design  Design  Design  Design  Design  Design  Design- Implementation description  Design  Population  Design  Design- Implementation description  Design- Implementation description  Design  Design  Design  Design  Design  Population  Design  Design- Implementation description  Design  Design  Design  Design  Design  Design  Design  Design  Design  Design  Design  Design- Implementation description  Design  Design  Design  Design  Design  Communicable disease  Design  Design- implementation description  Design  Design  Design  Intervention  Design  Communicable disease and design  Communicable disease and design  Design  Design  Design  Intervention  Intervention and design  Design  Design  Intervention and design  Design- Implementation description  Design  Design- implementation description  Design- implementation description  Design  Intervention and design  Design  Design  Design  Design  Population  Communicable disease  Intervention  Design – Implementation description  Design  Intervention  Population  Design  Design  Design  Communicable disease  Design  Design  Population  Design  Intervention  Outcomes  Communicable disease  Intervention  Communicable disease  Population  Design  Design  Intervention  Design  Population  Design  Design  Intervention  Design  Intervention  Intervention  Intervention  Design  Design  Design  Communicable disease  Design  Design- Implementation description  Population  Design  Intervention  Intervention  Design- Implementation description  Design  Intervention  Design  Intervention  Population  Intervention  Communicable disease  Communicable disease  Communicable disease  Communicable disease  Design  Design  Design- Implementation description  Communicable disease  Design  Design  Population  Intervention  Design  Design  Communicable disease  Communicable disease  Design  Intervention  Design  Intervention  Intervention  Intervention  Intervention  Communicable disease  Population  Design  Design  Design  Population  Intervention |

1. Bailey P, Binh H, Bang H. Promoting accountability in obstetric care: Use of criteria-based audit in Viet Nam. Global Public Health: An International Journal for Research, Policy and Practice. 2010;5(1):62-74.

2. Bailie R, Si D, Dowden M, O'Donoghue L, Connors C, Robinson G, et al. Improving organisational systems for diabetes care in Australian Indigenous communities. BMC Health Services Research. 2007;7(67).

3. Bailie RS, Si D, Robinson GW, Togni SJ, d'Abbs PHN. A multifaceted health-service intervention in remote Aboriginal communities: 3-year follow-up of the impact on diabetes care. Medical Journal of Australia. 2004;181(4):195-200.

4. Bain NSC, Foster K, Grimshaw J, MacLeod TN, Broom J, Reid J, et al. Can audit of a local protocol for the management of lipid disorders effect and detect a change in clinical practice? Health Bulletin. 1997;55(2):94-101.

5. Baker U, Tomson G, Some M, Kouyate B, Williams J, Mpembeni R, et al. 'How to know what you need to do': a cross-country comparison of maternal health guidelines in Burkina Faso, Ghana and Tanzania. Implementation Science. 2012;7:31.

6. Balsara ZP, Hussein MH, Winch PJ, Gipson R, Santosham M, Darmstadt GL. Impact of clean delivery kit use on clean delivery practices in Beni Suef Governorate, Egypt. Journal of Perinatology. 2009;29(10):673-9.

7. Bampton PA, Sandford JJ, Young GP. Achieving long-term compliance with colonoscopic surveillance guidelines for patients at increased risk of colorectal cancer in Australia. International Journal of Clinical Practice. 2007;61(3):510-3.

8. Banerjee SK, Andersen K, Jaydeep T, Mandakini P, Milind S, Uday T. Evaluation of a network of medical abortion providers in two districts of Maharashtra, India. Global Public Health. 2011;6(3):283-92.

9. Barcelo A, Cafiero E, de Boer M, Mesa AE, Lopez MG, Jimenez RA, et al. Using collaborative learning to improve diabetes care and outcomes: the VIDA project. Primary care diabetes. 2010;4(3):145-53.

10. Basit A, Khan A, Khan RA. BRIGHT Guidelines on Self-Monitoring of Blood Glucose. Pakistan Journal of Medical Sciences. 2014;30(5):1150-5.

11. Belaid L, Ridde V. An implementation evaluation of a policy aiming to improve financial access to maternal health care in Djibo district, Burkina Faso. BMC Pregnancy & Childbirth. 2012;12:143.

12. Beran D, Silva Matos C, Yudkin JS. The Diabetes UK Mozambique Twinning Programme. Results of improvements in diabetes care in Mozambique: a reassessment 6 years later using the Rapid Assessment Protocol for Insulin Access. Diabetic Medicine. 2010;27(8):855-61.

13. Beran D, Yudkin JS, Courten Md. Assessing health systems for type 1 diabetes in sub-Saharan Africa: developing a 'Rapid Assessment Protocol for Insulin Access'. BMC Health Services Research. 2006;6(17).

14. Berglund A, Lefevre-Cholay H, Bacci A, Blyumina A, Lindmark G. Successful implementation of evidence-based routines in Ukrainian maternities. Acta Obstetricia et Gynecologica Scandinavica. 2010;89(2):230-7.

15. Bernal C, Velasquez C, Alcaraz G, Botero J. Treatment of severe malnutrition in children: experience in implementing the World Health Organization guidelines in Turbo, Colombia. Journal of Pediatric Gastroenterology & Nutrition. 2008;46(3):322-8.

16. Bernardo WM. [Clinical guidelines in brazilian health system]. Revista Da Associacao Medica Brasileira. 2008;54(5):377.

17. Berra K, Ma J, Klieman L, Hyde S, Monti V, Guardado A, et al. Implementing cardiac risk-factor case management: lessons learned in a county health system. Critical Pathways in Cardiology: A Journal of Evidence-Based Medicine. 2007;6(4):173-9.

18. Berti A, Bregani ER, Manenti F, Pizzi C. Outcome of severely malnourished children treated according to UNICEF 2004 guidelines: a one-year experience in a zone hospital in rural Ethiopia. Transactions of the Royal Society of Tropical Medicine & Hygiene. 2008;102(9):939-44.

19. Bordon JG, Paiva SA, Matsubara LS, Inoue RM, Matsui M, Gut AL, et al. Mortality decline after implementation of standard protocols for treating patients with acute myocardial infarction. Arquivos Brasileiros de Cardiologia. 2004;82(4):370-7.

20. Boskabady MH, Rezaeitalab F, Rahimi N, Dehnavi D. Improvement in symptoms and pulmonary function of asthmatic patients due to their treatment according to the Global Strategy for Asthma Management (GINA). BMC Pulmonary Medicine. 2008;8:26.

21. Boucar M, Hill K, Coly A, Djibrina S, Saley Z, Sangare K, et al. Improving postpartum care for mothers and newborns in Niger and Mali: a case study of an integrated maternal and newborn improvement programme. BJOG: An International Journal of Obstetrics & Gynaecology. 2014;121 Suppl 4:127-33.

22. Bouchet B, Francisco M, Ovretveit J. The Zambia quality assurance program: successes and challenges. International Journal for Quality in Health Care. 2002;14 Suppl 1:89-95.

23. Bouchlaka A, Ben Abdallah M, Ben Aissa R, Smida S, Ouechtati A, Boussen H, et al. [Practice of large scale mammography in the Ariana area of Tunisia: prelude to a mass screening?]. Tunisie Medicale. 2009;87(7):426-31.

24. Boulet LP, FitzGerald JM, Levy ML, Cruz AA, Pedersen S, Haahtela T, et al. A guide to the translation of the Global Initiative for Asthma (GINA) strategy into improved care. European Respiratory Journal. 2012;39(5):1220-9.

25. Brooks GL. Improving the management of patients with type-2 diabetes in a rural clinic. Dissertation Abstracts International: Section B: The Sciences and Engineering. 2012;72(10-B):5880.

26. Brosseau L, Wells GA, Kenny GP, Reid R, Maetzel A, Tugwell P, et al. The implementation of a community-based aerobic walking program for mild to moderate knee osteoarthritis (OA): a knowledge translation (KT) randomized controlled trial (RCT): Part I: The Uptake of the Ottawa Panel clinical practice guidelines (CPGs). BMC Public Health. 2012;12(871).

27. Brusamento S, Legido-Quigley H, Panteli D, Turk E, Knai C, Saliba V, et al. Assessing the effectiveness of strategies to implement clinical guidelines for the management of chronic diseases at primary care level in EU Member States: a systematic review. Health Policy. 2012;107(2/3):168-83.

28. Casey GJ, Phuc TQ, Macgregor L, Montresor A, Mihrshahi S, Thach TD, et al. A free weekly iron-folic acid supplementation and regular deworming program is associated with improved hemoglobin and iron status indicators in Vietnamese women. BMC Public Health. 2009;9:261.

29. Cavalli-Sforza T. Effectiveness of weekly iron-folic acid supplementation to prevent and control anemia among women of reproductive age in three Asian countries: development of the master protocol and implementation plan. Nutrition Reviews. 2005;63(12 Pt 2):S77-80.

30. Cheah J. Development and implementation of a clinical pathway programme in an acute care general hospital in Singapore. International Journal for Quality in Health Care. 2000;12(5):403-12.

31. Cho I, Kim J, Kim JH, Kim HY, Kim Y. Design and implementation of a standards-based interoperable clinical decision support architecture in the context of the Korean EHR. International Journal of Medical Informatics. 2010;79(9):611-22.

32. Chowchuen B, Godfrey K. Development of a network system for the care of patients with cleft lip and palate in Thailand. Scandinavian Journal of Plastic & Reconstructive Surgery & Hand Surgery. 2003;37(6):325-31.

33. Cook CB, Stockton L, Baird M, Osburne RC, Davidson PC, Steed RD, et al. Working to improve care of hospital hyperglycemia through statewide collaboration: the Georgia Hospital Association Diabetes Special Interest Group. Endocrine Practice. 2007;13(1):45-50.

34. Dantas RA, Aguillar OM, dos Santos Barbeira CB. Implementation of a nurse-monitored protocol in a Brazilian hospital: a pilot study with cardiac surgery patients. Patient Education & Counseling. 2002;46(4):261-6.

35. de Carvalho FA, Schwamm LH, Kuster GW, Bueno Alves M, Cendoroglo Neto M, Sampaio Silva G. Get with the guidelines stroke performance indicators in a brazilian tertiary hospital. Cerebrovascular Diseases Extra. 2012;2(1):26-35.

36. de Souza CA. Five years of implementation of guidelines in hematology and transfusion medicine in Brazil. Revista Brasileira de Hematologia e Hemoterapia. 2014;36(3):165-6.

37. Deen JL, Funk M, Guevara VC, Saloojee H, Doe JY, Palmer A, et al. Implementation of WHO guidelines on management of severe malnutrition in hospitals in Africa. Bulletin of the World Health Organization. 2003;81(4):237-43.

38. Deen JL, Weber M, Qazi S, Fontaine O. An extended field test of the WHO severe malnutrition guidelines in hospitals in developing countries: preliminary observations. Forum of Nutrition. 2003;56:183-4.

39. Deng Y, Jiao Y, Hu R, Wang Y, Wang Y, Zhao X. Reduction of length of stay and costs through the implementation of clinical pathways for stroke management in China. Stroke. 2014;45(5):e81-3.

40. Drost E, van Lonkhuijzen LR, Meguid T, Landis Lewis D, Zeeman GG. Implementing safe motherhood: a low-cost intervention to improve the management of eclampsia in a referral hospital in Malawi. BJOG: An International Journal of Obstetrics & Gynaecology. 2010;117(12):1553-7.

41. du Mortier S, Arpagaus M. Quality improvement programme on the frontline: an International Committee of the Red Cross experience in the Democratic Republic of Congo. International Journal for Quality in Health Care. 2005;17(4):293-300.

42. Echeverri C, Castilla J. A case study in Columbia: Implementation of the IASC Guidelines on Mental Health and Psychosocial Support in Emergency Settings. Intervention: International Journal of Mental Health, Psychosocial Work & Counselling in Areas of Armed Conflict. 2008;6(3-4):284-90.

43. English M, Wamae A, Nyamai R, Bevins B, Irimu G. Implementing locally appropriate guidelines and training to improve care of serious illness in Kenyan hospitals: a story of scaling-up (and down and left and right). Archives of Disease in Childhood. 2011;96(3):285-90.

44. Falbo AR, Alves JG, Batista Filho M, Cabral-Filho JE. [Implementation of World Health Organization guidelines for management of severe malnutrition in a hospital in Northeast Brazil]. Cadernos de Saude Publica. 2006;22(3):561-70.

45. Fang X, Li S, Gao L, Zhao N, Wang X, Bai C. A short-term educational program improved physicians' adherence to guidelines for COPD and asthma in Shanghai. Clinical and Translational Medicine. 2012;1(1):13.

46. Franco LM, Marquez L. Effectiveness of collaborative improvement: evidence from 27 applications in 12 less-developed and middle-income countries. BMJ Quality & Safety. 2011;20(8):658-65.

47. Fuzinatto F, Waldemar FS, Wajner A, Elias CA, Fernandez JF, Hopf JL, et al. A clinical decision support system for venous thromboembolism prophylaxis at a general hospital in a middle-income country. Jornal Brasileiro De Pneumologia: Publicacao Oficial Da Sociedade Brasileira De Pneumologia E Tisilogia. 2013;39(2):138-46.

48. Gill JM, Ross A, Pirie F, Esterhuizen T. The effect of the introduction of a standard monitoring protocol on the investigations performed on the metabolic control of type 2 diabetes at Addington Hospital Medical Outpatients Department, Durban, South Africa. South African Family Practice. 2012;54(2):151-5.

49. Goldhaber-Fiebert JD, Denny LA, De Souza M, Kuhn L, Goldie SJ. Program spending to increase adherence: South African cervical cancer screening. PLoS ONE [Electronic Resource]. 2009;4(5):e5691.

50. Govender I, Ehrlich R, Van Vuuren U, De Vries E, Namane M, De Sa A, et al. Clinical audit of diabetes management can improve the quality of care in a resource-limited primary care setting. International Journal for Quality in Health Care. 2012;24(6):612-8.

51. Gulliford MC, Mahabir D. A five-year evaluation of intervention in diabetes care in Trinidad and Tobago. Diabetic Medicine. 1999;16(11):939-45.

52. Gulmezoglu AM, Villar J, Grimshaw J, Piaggio G, Lumbiganon P, Langer A. Cluster randomized trial of an active, multifaceted information dissemination intervention based on The WHO Reproductive health library to change obstetric practices: methods and design issues [ISRCTN14055385]. BMC Medical Research Methodology. 2004;4:2.

53. Ider BE, Adams J, Morton A, Whitby M, Muugolog T, Lundeg G, et al. Using a checklist to identify barriers to compliance with evidence-based guidelines for central line management: a mixed methods study in Mongolia. International Journal of Infectious Diseases. 2012;16(7):e551-7.

54. Irimu GW, Gathara D, Zurovac D, Kihara H, Maina C, Mwangi J, et al. Performance of health workers in the management of seriously sick children at a Kenyan tertiary hospital: before and after a training intervention. PLoS ONE [Electronic Resource]. 2012;7(7):e39964.

55. Irimu GW, Greene A, Gathara D, Kihara H, Maina C, Mbori-Ngacha D, et al. Explaining the uptake of paediatric guidelines in a Kenyan tertiary hospital--mixed methods research. BMC Health Services Research. 2014;14:119.

56. Jamsek VV. The protocol of chronic patient management in a family medicine practice. [Slovenian]. Zdravniski Vestnik. 2013;82(11):711-7.

57. Jin Y, Ma H. Development and implementation of clinical pathways in dermatology. [Chinese]. Chinese Journal of Dermatovenereology. 2011;25(2):140-1.

58. Jordans M, Upadhayab N, Tol W, Shrestha P, Douce J, Gurung R, et al. Introducing the IASC Mental Health and Psychsocial Support Guidelines in Emergencies in Nepal: A process description. Intervention: International Journal of Mental Health, Psychosocial Work & Counselling in Areas of Armed Conflict. 2010;8(1):52-63.

59. Joyner K, Mash B. A comprehensive model for intimate partner violence in South African primary care: action research. BMC Health Services Research. 2012;12:399.

60. Kamala K. Developing and implementing dietary guidelines in India. Asia Pacific Journal of Clinical Nutrition. 2008;17(Supplement 1):66-9.

61. Kan XH, Chiang CY, Enarson DA, Rao HL, Chen Q, Ait-Khaled N, et al. Asthma as a hidden disease in rural China: opportunities and challenges of standard case management. Public Health Action. 2012;2(3):87-91.

62. Karrowni W, Abdallah M, Itani S, Kobeissi L, Shamseddeen W, Dakik HA. Management of acute coronary syndromes in developing countries: are we complying with practice guidelines? International Journal of Cardiology. 2010;144(1):95-6.

63. Kathawaroo S, Hukins G. Asthma management in practice. South African Medical Journal Suid-Afrikaanse Tydskrif Vir Geneeskunde. 2004;94(10):832-3.

64. Kengne AP, Fezeu L, Sobngwi E, Awah PK, Aspray TJ, Unwin NC, et al. Type 2 diabetes management in nurse-led primary healthcare settings in urban and rural Cameroon. Primary care diabetes. 2009;3(3):181-8.

65. Klein JD, Allan MJ, Elster AB, Stevens D, Cox C, Hedberg VA, et al. Improving adolescent preventive care in community health centers. Pediatrics. 2001;107(2):318-27.

66. Kongnyuy EJ, Mlava G, van den Broek N. Using criteria-based audit to improve the management of postpartum haemorrhage in resource limited countries: a case study of Malawi. Maternal & Child Health Journal. 2009;13(6):873-8.

67. Kuhne F, Haagen M, Baldus C, Diareme S, Grether A, Schmitt F, et al. Implementation of preventive mental health services for children of physically ill parents: Experiences in seven European countries and health care systems. General Hospital Psychiatry. 2013;35(2):147-53.

68. Kupczyk M, Haahtela T, Cruz AA, Kuna P. Reduction of asthma burden is possible through National Asthma Plans. Allergy. 2010;65(4):415-9.

69. Kwast BE. Reduction of maternal and perinatal mortality in rural and peri-urban settings: what works? European Journal of Obstetrics, Gynecology, & Reproductive Biology. 1996;69(1):47-53.

70. Lalonde AB, Grellier R. FIGO saving mothers and newborns initiative 2006-2011. International Journal of Gynaecology & Obstetrics. 2012;119 Suppl 1:S18-21.

71. Latham T, Malomboza O, Nyirenda L, Ashford P, Emmanuel J, M'Baya B, et al. Quality in practice: implementation of hospital guidelines for patient identification in Malawi. International Journal for Quality in Health Care. 2012;24(6):626-33.

72. Lima SM, Portela MC, Koster I, Escosteguy CC, Ferreira VM, Brito C, et al. [Use of clinical guidelines and the results in primary healthcare for hypertension]. Cadernos de Saude Publica. 2009;25(9):2001-11.

73. Liwsrisakun C, Pothirat C. Actual implementation of the Thai Asthma Guideline. Journal of the Medical Association of Thailand. 2005;88(7):898-902.

74. Loch A, Lwin T, Zakaria IM, Abidin IZ, Wan Ahmad WA, Hautmann O. Failure to improve door-to-needle time by switching to emergency physician-initiated thrombolysis for ST elevation myocardial infarction. Postgraduate Medical Journal. 2013;89(1052):335-9.

75. Lopes SC, Cabral AJ, de Sousa B. Community health workers: to train or to restrain? A longitudinal survey to assess the impact of training community health workers in the Bolama Region, Guinea-Bissau. Human Resources for Health [Electronic Resource]. 2014;12:8.

76. Lundgren R, Sinai I, Jha P, Mukabatsinda M, Sacieta L, Leon FR. Assessing the effect of introducing a new method into family planning programs in India, Peru, and Rwanda. Reproductive Health. 2012;9:17.

77. Luz AG, Osis MJ, Ribeiro M, Cecatti JG, Amaral E. Impact of a nationwide study for surveillance of maternal near-miss on the quality of care provided by participating centers: a quantitative and qualitative approach. BMC Pregnancy & Childbirth. 2014;14:122.

78. Moran AC, Kerber K, Pfitzer A, Morrissey CS, Marsh DR, Oot DA, et al. Benchmarks to measure readiness to integrate and scale up newborn survival interventions. (Special Issue: A decade of change for newborn survival, policy and programmes (2000-2010): A multi-country evaluation of progress towards scale.). Health Policy and Planning. 2012;27(Suppl. 3).

79. Mottaghipour Y, Salesian N, Seddigh A, Roudsari MJ, Hosseinzade ST, Sharifi V. Training health professionals to conduct family education for families of patients with first-episode psychosis: adherence to protocol. Iranian Journal of Psychiatry. 2010;5(1):7-10.

80. Mousavi M, Hayatshahi A, Sarayani A, Hadjibabaie M, Javadi M, Torkamandi H, et al. Impact of clinical pharmacist-based parenteral nutrition service for bone marrow transplantation patients: a randomized clinical trial.[Erratum appears in Support Care Cancer. 2013 Dec;21(12):3449]. Supportive Care in Cancer. 2013;21(12):3441-8.

81. Mshelia C, Huss R, Mirzoev T, Elsey H, Baine SO, Aikins M, et al. Can action research strengthen district health management and improve health workforce performance? A research protocol. BMJ Open. 2013;3(8):e003625.

82. Mwakyusa S, Wamae A, Wasunna A, Were F, Esamai F, Ogutu B, et al. Implementation of a structured paediatric admission record for district hospitals in Kenya--results of a pilot study. BMC International Health & Human Rights. 2006;6:9.

83. Mwaniki MK, Vaid S, Chome IM, Amolo D, Tawfik Y, Kwale Improvement C. Improving service uptake and quality of care of integrated maternal health services: the Kenya Kwale District improvement collaborative. BMC Health Services Research. 2014;14:416.

84. Mwita CC, Akello W, Sisenda G, Ogoti E, Tivey D, Munn Z, et al. Assessment of cardiovascular risk and target organ damage among adult patients with primary hypertension in Thika Level 5 Hospital, Kenya: a criteria-based clinical audit. International Journal of Evidence-Based Healthcare. 2013;11(2):115-20.

1. Mwaniki P, Ayieko P, Todd J and English M. Assessment of paediatric inpatient care during a multifaceted quality improvement intervention in Kenyan district hospitals--use of prospectively collected case record data. BMC Health Services Research 2014 14: 312
2. Naimoli J F, Rowe A K, Lyaghfouri A, Larbi R and Lamrani L A. Effect of the Integrated Management of Childhood Illness strategy on health care quality in Morocco. International Journal for Quality in Health Care 2006 18(2): 134-144.
3. Nausheen S, Hammad R, Khan A Rational use of antibiotics — a quality improvement initiative in hospital setting Journal of the Pakistan Medical Association 2013 63(1)
4. Navarro VR, Falcón A, Iraola MD, Valladares F, Ordúñez P O. Reducing case fatality from acute myocardial infarction in Cienfuegos, Cuba, 1994-2009. MEDICC Review 2012 14(4): 14-18
5. Navipour H, Nayeri N, Hooshmand A, and Zargar M T. An investigation into the effects of quality improvement method on patients' satisfaction: a semi experimental research in Iran." Acta Medica Iranica 2011 49(1): 38-43.
6. Nzinga J, Ntoburi S, Wagai J,, Mbindyo P, Mbaabu L, Migiro S, Wamae A, Grace Irimu G,and English M. Implementation experience during an eighteen month intervention to improve paediatric and newborn care in Kenyan district hospitals. Implementation Science 2009 4: 45.
7. Nsimba SED, Assessing the impact of educational intervention for improving management of malaria and other childhood illnesses in Kibaha District-Tanzania. East African Journal of Public Health 2007 4(1): 5-11.
8. Nutley T, Gnassou L, Traore M, Abitche Edwige Bosso AE and Mullen S. Moving data off the shelf and into action: an intervention to improve data-informed decision making in Cote d'Ivoire. Global Health Action 2014 7: 25035.
9. Obua C, Ogwal-Okeng J W, Waako P,Aupont O and Ross-Degnan D Impact of an educational intervention to improve prescribing by private physicians in Uganda. East African Medical Journal 2004 81(2) Suppl: S17-24.
10. Oliver-Vazquez M, Sanchex-Ayendez M, Suarez-Perez E, Velez-Almodovar H, Arroy-Calderon Y. Breast cancer health promotion model for older Puerto Rican women: results of a pilot programme. Health Promotion International 2002 17(1): 3-11.
11. Onat A, Soydan, I., Tokgozoglu, L, Sansoy, V, Koylan N, Domanic, N., Dilek Ural, D Guideline implementation in a multicenter study with an estimated 44% relative cardiovascular event risk reduction. Clinical Cardiology 2003 26(5): 243-249.
12. Ostojic MC and. Karanovic ND Mentality and organisational changes are key to developing primary angioplasty Eurointervention 2012 8 86-89
13. Omaswa F, Burnham G, Baingana G, Mwebesa H and Morrow R. Introducing quality management into primary health care services in Uganda. Bulletin of the World Health Organization, 1997, 75 (2): 155-161
14. Osur J, Baird T L, Levandowski B A, Jackson E and Murokora D. Implementation of misoprostol for postabortion care in Kenya and Uganda: a qualitative evaluation. Global Health Action 2013, 6**:** 19649
15. Ozgun H, Ertugrul B M, Soyder A, Ozturk B, Aydemir M. Peri-operative antibiotic prophylaxis: adherence to guidelines and effects of educational intervention. International Journal of Surgery 2010 8(2): 159-163.
16. Patel P, Weiss H A, Chowdhary N, Naik S, Pednekar S, Chatterjee S, De Silva M J, Bhat B, Araya R, King M, Simon G, Verdeli H and Kirkwood B R. Effectiveness of an intervention led by lay health counsellors for depressive and anxiety disorders in primary care in Goa, India (MANAS): a cluster randomised controlled trial. The Lancet 2010 376: 2086–95
17. Paulik E, Muller A, Belicza E, Boda K and Nagymajtenyi L. Use of echocardiography among patients with heart failure: practice variations in Hungarian hospitals. International Journal for Quality in Health Care 2002 14(4): 313-319.
18. Pedalini L M, Gandlfi Dallari S, and Barber-Madden R. Public Health Advocacy on Behalf of Women in Sao Paulo: Learning to Participate in the Planning Process. Journal of Public Health Policy 1993 14 (2) 183-197
19. Peng B, Ni J, Anderson CS, Zhu Y, Wang Y, Pu C, Wu J, Wang J; Zhou L, Yao M, He J, Shan G, Gao, S Xu W, Cui L. Implementation of a structured guideline-based program for the secondary prevention of ischemic stroke in China. Stroke 2014 45(2): 515-519.
20. Penny M W, Creed-Kanashiro H M, Robert R C, Narro M R, Laura E Caulfield L E and Black R E. Effectiveness of an educational intervention delivered through the health services to improve nutrition in young children: a cluster-randomised controlled trial Lancet 2005; 365: 1863–72
21. Perez Villasante LP, Raigada Mares J, Collins Estrada A, Alza SM, Parodi AF, Jiménez Castro S, Casas Castañeda J Effectiveness of an educational program focusing in healthy lifestyles for overweight and obesity reduction in Robert M. Smith School, Huaraz, Ancash, Peru Acta Medica Peruana 2008 25(4): 204-209
22. Perichart-Perera O, Balas-Nakash M, Parra-Covarrubias A, Rodriguez-Cano A, Ramirez-Torres A, Ortega-González C, and Vadillo-Ortega F A medical nutrition therapy program improves perinatal outcomes in Mexican pregnant women with gestational diabetes and type 2 diabetes mellitus. Diabetes Educator 2009 35(6): 1004-1013.
23. Phassarang C, Wahlström R, Phoummalaysith B, Boupha B and Tomson G. Building the national drug policy on evidence--a cross sectional study on assessing implementation in Lao PDR. Southeast Asian Journal of Tropical Medicine & Public Health 2002 33(3): 647-653.
24. Phed-on U, Naowapanich S, Poolsawat U, Nimmannit A and Wongpraparut N. Benefit of post PCI medical checklist to improve adhering with best practice guidelines in the patients with coronary artery disease undergoing percutaneous coronary intervention (PCI). Journal of the Medical Association of Thailand 2011 94 Suppl 1: S1-10.
25. Phuc TQ, Mihrshahi S, CaseyGJ, Phu LB, Tien NT, Caruana SR, Thach TD, Montresor A and Biggs B-A Lessons learned from implementation of a demonstration program to reduce the burden of anemia and hookworm in women in Yen Bai Province, Viet Nam BMC Public Health 2009 **9**:266
26. Pinto MCFG, Bueno AC and Vieira A A. Implementation of a protocol proposed by the Brazilian National Health Surveillance Agency for antibiotic use in very low birth weight infants. Jornal de Pediatria 2013 89(5): 450-455
27. Prata N, Quaiyum A, Passano P, Bell S, Bohl D, Hossain S, Azmi AJ, Begum M. Training traditional birth attendants to use misoprostol and an absorbent delivery mat in home births 2012 Social Science & Medicine 75 2012 2021-2027
28. Pumprueg S, Chotinaiwattarakul C, Nopmaneejumruslers C , Nimmannit A, Wiratchpintu P, Charernthai S. Primary percutaneous coronary intervention in acute myocardial infarction at Siriraj Hospital: the improvement over time. Journal of the Medical Association of Thailand 2007 90 Suppl 2: 19-24.
29. Queiroz, R, Grinbaum R S, Galvão L L, Tavares F G and Bergsten-Mendes G. Antibiotic prophylaxis in orthopedic surgeries: the results of an implemented protocol. Brazilian Journal of Infectious Diseases 2005 9(4): 283-287
30. Rahimi-Rad M H, SeidSalehi S Improvement of venous thromboembolism prophylaxis by attaching printed thrombosis risk assessment tool and recommendations to patients hospital charts 2010 Pneumologia 59(3) 140-143
31. Rahman A, Malik A, Sikander S, Roberts C and Creed F. Cognitive behaviour therapy-based intervention by community health workers for mothers with depression and their infants in rural Pakistan: a cluster-randomised controlled trial. The Lancet 2008 372: 902-909
32. Ramezankhani, A., Mirmiran P and Azizi F. Effect of nutritional intervention on the prevalence of metabolic syndrome and heart disease risk factors in urban Tehran (Tehran lipid and glucose study). Eastern Mediterranean Health Journal 2011 17(6): 501-508.
33. Ramírez Prada D, Delgado G, Hidalgo Patiño, Pérez-Navero J and Gil Campos m G. Using of WHO guidelines for the management of severe malnutrition to cases of marasmus and kwashiorkor in a Colombia children's hospital." Nutricion Hospitalaria 2011 26(5): 977-983.
34. Ratanalert S, Kornsilp T, Chintragoolpradub N, Kongchoochouy S.,The impacts and outcomes of implementing head injury guidelines: clinical experience in Thailand Emergency Medicine Journal 2007;24:25–30.
35. Rawat R, Nguyen P H, Ali D, Saha K, Alayon S, Kim S S, Ruel M, and Purnima Menon P. Learning how programs achieve their impact: embedding theory-driven process evaluation and other program learning mechanisms in Alive & Thrive. (Special Issue: Designing large-scale programs to improve infant and young child feeding in Asia and Africa: methods and lessons of Alive & Thrive). Food and Nutrition Bulletin 2013 34(Suppl. 2): S212-S225.
36. Riachy, D M, Safi J, Chalouhy G, Andari J, and Khayat G.. (2010). Application of a hospital audit to the use of nebulizers: the Case of the Hotel-Dieu de France-Lebanon. Revue des Maladies Respiratoires 2010 27(9): 1049-1054
37. Ribeiro A G, Ribeiro S M R, Dias C M G C, Ribeiro A Q, Castro F A F, Suárez-Varela M M and Cotta R M M. Non-pharmacological treatment of hypertension in primary health care: a comparative clinical trial of two education strategies in health and nutrition. BMC Public Health 2011 11(637).
38. Ristić S, Miljković B, Vezmar S Stanojević D Are local clinical guidelines useful in promoting rational use of antibiotic prophylaxis in Caesarean delivery? Pharmacy World & Science 2010 32 139–145
39. Robert R C, Gittelsohn J, Creed-Kanashiro H M, Penny M E, Caulfield L E, Narro M R, Steckler A and Black R E. Implementation examined in a health center-delivered, educational intervention that improved infant growth in Trujillo, Peru: successes and challenges. Health Education Research 2007 22(3): 318-331.
40. Ross-Degnan D, Soumerai SB, Goel PK, Bates J, Makhulo J, Dondi N, Sutoto, Adi D, Ferraz-Tabor L and Hogan R. The impact of face-to-face educational outreach on diarrhoea treatment in pharmacies. Health Policy and Planning 1996 11(3): 308-318
41. Rowe A K, Onikpo F, Lama M, Osterholt D M, Rowe S Y, and Deming M S. A multifaceted intervention to improve health worker adherence to integrated management of childhood illness guidelines in Benin. American Journal of Public Health 2009 99(5): 837-846.
42. Rowe, A K, Osterholt D M, Kouame J, Piercefield E, Herman K M, Onikpo F, Lama M, Deming M S. Trends in health worker performance after implementing the Integrated Management of Childhood Illness strategy in Benin. Tropical Medicine & International Health 2012 17(4): 438-446
43. Saval K, Izham M, Ibrahim M, Shankar R P, Palaian S and Mishra P. Evaluation of academic detailing programme on childhood diarrhoea management by primary healthcare providers in Banke district of Nepal. Journal of Health, Population and Nutrition 2013 31(2): 231-242.
44. Schilperoord S, Buffoni L and Kouyou W. UNHCR’s potential and its challenges in implementing the IASC Guidelines on Mental Health and Psychosocial Support in Emergency Settings in the Ethiopia context. International Journal of Mental Health, Psychosocial Work & Counselling in Areas of Armed Conflict 2008 6(3-4): 307-309.
45. Sharif-Kashani B, Raeissi S, Bikdeli B, Shahabi P, Behzadnia N, Saliminejad L, Samiei-Nejad M, Nasiri F, Khayyami M, Forootan B, Pozhan S, Masjedi M-R. Sticker reminders improve thromboprophylaxis appropriateness in hospitalized patients Thrombosis Research 2010 126 211–216
46. Sheik L, Najmi N, Khalid U and Saleem T. Evaluation of compliance and outcomes of a management protocol for massive postpartum hemorrhage at a tertiary care hospital in Pakistan. BMC Pregnancy & Childbirth 2011 11: 28
47. Silva J.M., Stein A.T, Schünemann H.J., Bordin R, Kuchenbecker R,.Drachler ML & Lourdes Drachler M. Academic detailing and adherence to guidelines for Group B streptococci prenatal screening: a randomized controlled trial BMC Pregnancy & Childbirth 2013, 13:68
48. Siow J Y, Mei Lai P S, Siew Siang Chua S S and Chan S P. The impact of pharmacist intervention on the use of activated vitamin D in a tertiary referral hospital in Malaysia. International Journal of Pharmacy Practice 2009 17 pp 305-311
49. Smith H, Brown H, Hofmeyr G. J, Garner P. Evidence-based obstetric care in South Africa — influencing practice through the ‘Better Births Initiative’ South African Medical Journal 2004, 94(2) 117-120
50. Stanback J, Griffey S, Lynam P, Ruto C and Cummings S, Improving adherence to family planning guidelines in Kenya: an experiment. International Journal for Quality in Health Care 2007 19(2): 68-73.
51. Sun J and Stewart D Resilience and depression in children: Mental health promotion in primary schools in China. International Journal of Mental Health Promotion 2007 9(4): 37-46.
52. Tan A and Aït-Khaled N. Dissemination and implementation of guidelines for the treatment of asthma. International Journal of Tuberculosis & Lung Disease 2006 10(7): 710-716.
53. Ten Asbroek A, Delnoij DMJ, Niessen L W, ScherpbierR W, Shrestha N, Bam D S, Gunnerberg C, CW Van Der Hor C W and Klazinga N S. Implementing global knowledge in local practice: a WHO lung health initiative in Nepal. Health Policy & Planning 2005 20(5): 290-301.
54. Thamlikitkul, V. and W. Apisitwittaya Implementation of clinical practice guidelines for upper respiratory infection in Thailand. International Journal of Infectious Diseases 2004 8(1): 47-51.
55. Thiam S, Thwing J, Diallo I, Fall FB, Diouf MB, Perry R, Medoune Ndiop M, Diouf ML, Cisse MM, Diaw MM and Moussa Thior M Scale-up of home-based management of malaria based on rapid diagnostic tests and artemisinin-based combination therapy in a resource-poor country: results in Senegal 2012 Malaria Journal 11 334
56. Tohtubtiang K and Tantibhaedhyangkul U. Using risk-assessment sheet to improve effectiveness of health examination service. Journal of the Medical Association of Thailand 2005 88 Suppl 3: S63-70.
57. Trap B, Todd C H, Moore H and Laing R. The impact of supervision on stock management and adherence to treatment guidelines: a randomized controlled trial. Health Policy & Planning 2001 16(3): 273-280.
58. Trostler N, Myers E, Alphan E, Endvelt R, Voet H, Outcomes Monitoring and Implementing Evidence-Based Nutrition Practice Guidelines for Type 2 Diabetes Mellitus in 2 Middle Eastern Countries Topics in Clinical Nutrition 2013 28(3). 233–248
59. van de Vijver S, Otia S, Addoc J, de Graft-Aikins A and Agyemang C. Review of community-based interventions for prevention of cardiovascular diseases in low- and middle-income countries. Ethnicity & Health 2012 17(6): 651-676.
60. Verdeli H, Clougherty K, Onyango G, Lewandowski E, Speelman L, Betancourt T S, Neugebauer R, Stein T R and Bolton P. Group Interpersonal Psychotherapy for Depressed Youth in IDP Camps in Northern Uganda: Adaptation and Training. UNHCR’s potential and its challenges Intervention 2008, Volume 6, Number 3/4, 307 – 309
61. Verdeli H, Clougherty K, Bolton P, Speelman L, Ndogoni L , Bass J, Neugebauer R and. Weissman M M. Adapting group interpersonal psychotherapy for a developing country: experience in rural Uganda. World Psychiatry 2003 2:2
62. Visser L E, Kyei-Faried S, Belcher D W. Protocol and monitoring to improve snake bite outcomes in rural Ghana Transactions of the Royal Society of Tropical Medicine and Hygiene 2004 98 278—283
63. Wahlstrom R, Kounnavong S, Sisounthone B, Phanyanouvong A, Southammavong T, Eriksson B and Tomson G. Effectiveness of feedback for improving case management of malaria, diarrhoea and pneumonia – a randomized controlled trial at provincial hospitals in Lao PDR Tropical Medicine and International Health 2003 8 (10) 901–909
64. Weinburg M, Fuentes JM, Ruiz AI., Lozano F W, Angel E, Gaitan H, Goethe B, Parra S, Hellerstein S, Ross-Degnan D, Goldmann DA and Huskins C Reducing infections among women undergoing cesarean section in Colombia by means of continuous quality improvement methods. Archives of Internal Medicine 2001 161(19): 2357-2365.
65. Xiao D, Wang C, Chen H, and Hajek P. Making Hospitals in China Smoke-Free: A Prospective Study of Implementing the New Standard Nicotine & Tobacco Research 2013 15 (12) 2076–2080
66. Xu T, Wang H, Gong L, Ye H, Yu R, Wang D, Wang L, Feng Q, Chong Lee H, McGowan J E and Zhang T. The impact of an intervention package promoting effective neonatal resuscitation training in rural China. Resuscitation 2014 85(2): 253-259.
67. Xu Y, Ren X, Shi W & Jiang H. Implementation of the best practice in nasogastric tube feeding of critically ill patients in a neurosurgical intensive care unit International Journal of Evidence Based Healthcare 2013; 11**:** 128–133
68. Yang Z, Zhao P, Wang J, Tong L, Cao J, Tian Y, Yao Z, Wang J, Zhu Y, Jia Y, Wen A. DRUGS System Enhancing Adherence of Chinese Surgeons to Antibiotic Use Guidelines during Perioperative Period. PLoS ONE 2014 9(8): e102226.
69. Yu J, Roberts M, Shen Y and Wong M, Behavioral Family Therapy for Chinese Preschoolers with Disruptive Behavior: A Pilot Study. Journal of Child and Family Studies 2015 24:1192–1202
